# States, humanitarian advocacy, and crisis: persuasion, mobilization, and denunciation

**DOI:** 10.1186/s41018-026-00213-3

**Published:** 2026-10-22

**Authors:** Tom Ansell

**Affiliations:** The Hague Humanitarian Studies Centre, International Institute of Social Studies, Erasmus University Rotterdam, The Hague, Netherlands

**Keywords:** Humanitarian advocacy, Advocacy, Human rights, Slovenia, Colombia, Palestine, International Humanitarian Law

## Abstract

In times where humanitarian action is coming under pressure and attack, it is necessary to critically re-examine who carries out various humanitarian actions, such as advocacy, and how. This article aims to bring together several convergent themes to build a case for States to be seen to carry out ‘humanitarian advocacy’. The main question guiding this study is: “How, and in what ways do states utilize advocacy techniques, to affect humanitarian outcomes?” To approach this question, I examine humanitarian advocacy as a trend that runs alongside humanitarian diplomacy, as various factors contribute to humanitarian interactions occurring more often in the advocacy space. These includes ailing multilateral institutions, widescale flouting of International Humanitarian Law, and an appreciation of the complex interplay between a wide range of actors that carry out humanitarian action in some way, including states themselves (often ignored as humanitarian actors, in spite of their legal position as sovereign actors responsible for their populations). In an era of resilience humanitarianism, the role of advocacy as a humanitarian function has grown, and who is understood to be a humanitarian has similarly expanded. The discussion is supplemented by analysis of the official statements, actions, and policies of two less-powerful countries in the wake of the ongoing war on Gaza; the Republic of Colombia and the Republic of Slovenia. Three initial categorisations of ways in which states do humanitarian advocacy are offered, following analysis of the documentation, to serve as a basis for further study.

## Introduction

Why is advocacy for humanitarian purposes interesting to look at in a time where the (international) humanitarian sector is seen to be in crisis? Previously, (international) humanitarian organisations, supported by general respect for International Humanitarian Law by a majority of states, could provide relief for affected people, often in a delineated space negotiated between warring parties, or negotiated with a state to assist after a disaster. But many of the assumptions that underpinned this working relationship are crumbling: more states and groups flaunt International Humanitarian Law, multilateral institutions are paralysed, and there is a growing appreciation that carrying out humanitarian action is not exclusively the preserve of international humanitarian actors kowtowing to classical notions of mission and value. One of the implications of these changing realities is the growing role of states as humanitarian actors (Gomez, 2025). In the shadow of these large trends, and hitherto little analyzed, there is a shift in humanitarian advocacy practices. Formal humanitarian diplomacy has been joined by a widening of humanitarian advocacy, and more interactions between parties in a humanitarian context take place outside of official channels, and instead in the public area of advocacy. ‘Humanitarian advocacy’ is being employed to concentrate on a wider array of outcomes (Hilhorst & van Wessel 2022) as compared to ‘humanitarian diplomacy’ being mainly focused on humanitarian access, focusing on leaders, and relying on respect for international law (De Lauri, 2018).

This article aims to shed light on the use of advocacy to try and ensure international accountability and the protection of human rights (Metcalfe-Hough 2021). The wider context for this discussion comes from a general move within the humanitarian sector for change and reform ‘from below’ (Hilhorst et al. 2025) where changing currents in humanitarian aid provision are seen to emanate as much from affected societies and national institutions themselves. (Tabak 2016; Egger 2023; McLean & Hofman 2023). Disrespect for IHL, the need to interact with non-state stakeholders, and multilateral paralysis create conditions where even states must enter the public advocacy realm to affect outcomes such as humanitarian protection or access.

I identify three main areas, or techniques of how states might go about advocating: Public Advocacy (usually by official government representatives), Legal Advocacy (including convening and utilizing the international courts, or universal jurisdiction principles), and ‘Policy Advocacy’ – or ‘leading by example’. To illustrate these, I analyse the public statements, policies, legal approaches, and policy initiatives of two countries, Colombia and Slovenia, during the Israeli War on Gaza from October 2023 until now. These states are both working as active humanitarian advocates; with their Presidents and senior politicians regularly utilizing their positions and platforms to make public statements that draw attention to and plead the cause of affected people, through their convening of legal groupings and support of cases around International Humanitarian Law violation at the International Criminal Court, and their own government policies. States utilizing humanitarian aid to advance foreign policy interests is in no way a new trend (Apodaca 2017; Almezaini 2012; De Lauri 2016). However, the shifting geopolitical context of the 21 st Century makes this a timely moment to consider the ways and ends of how any why states may use humanitarian advocacy techniques.

The main question guiding this study is: “How do states utilize advocacy techniques, to do humanitarian advocacy?” My analytical frame consists of several approaches and trends from across Humanitarian Studies, International Relations, and Development Studies. This approach has been developed from a literature review, using a snowballing technique and guided by the following sub-questions: “What is the role of the state as a humanitarian actor?”, “What is humanitarian advocacy, and how does it relate to human rights advocacy, and humanitarian diplomacy?”.

The article has the following order: firstly, it discusses the understanding of humanitarianism and humanitarian advocacy that underpins this study. The article then approaches the topic of the state as a humanitarian actor: an oft-ignored role that states can and do play. Then, the relation between humanitarian advocacy and humanitarian diplomacy is explored, along with the conditions that have led to more and more humanitarian actions taking place in the realm of humanitarian advocacy rather than humanitarian diplomacy. This is then related to the relationship between humanitarian advocacy, and ‘resilience humanitarianism’ (Hilhorst 2018). Finally, I present the conditions under which even some states now need to utilize the advocacy space rather than the diplomatic space to affect humanitarian outcomes. After this literature review, I’ll look at some ways that states can be humanitarian advocates, and the techniques that they might deploy through analysing the actions and statements of two states-as-Humanitarian-Advocates: Slovenia and Colombia, both of which have deployed several humanitarian advocacy techniques during and throughout the period following the October 7, 2023, attacks on Israel, that precipitated the genocidal war on Gaza, as well as speaking out more generally in the face of polycrises and conflict.

## Understandings of humanitarianism and humanitarian crisis

A specific understanding of humanitarianism and humanitarian crisis underpins this discussion. I utilize the “arena” understanding (Hilhorst & Jansen 2010) of humanitarian contexts, where “the kinds of actions or actors considered to be humanitarian are not predetermined nor are the principles that qualify as humanitarian established in advance; instead I ask how the conditions of service delivery in crisis situations are shaped in practice” (Hilhorst & Pereboom 2015). A ‘humanitarian context’ is also akin to an “arena” (Hilhorst & Jansen 2010) in the socio-political sense, a space where “strategic groups equipped with their own logics and action strategies confront each other” (Blundo & Le Meur 2008, p. 12). In this frame of analysis, humanitarian response and service delivery is a result of negotiations and settlements between multiple actors. This includes formal humanitarian organizations, affected people, national and local civil society organizations, solidarity-based organizations, private companies, and the state in its national, regional, and local form(s) (see: Baguoi, 2021).

Interestingly, the “arena” approach may equally serve as a frame for looking at the current geopolitical milieus. The relationships between states in the ‘foreign policy arena’ (Webber & Smith 2014) can be best conceptualized in terms of the ‘complex interdependence theory’ (Keohane & Nye 2012), which holds that states and societies within the international system are characterized by multiple channels of linkage, that states’ and polities’ agendas are “not arranged in a clear or consistent hierarchy” (ibid, p. 20). States are free to define and decide in which ways and through which negotiations and settlements they will try to advance their various interests.

Humanitarian advocacy, that is “the activities of affected communities and their advocates to articulate, advance, and protect their rights (i.e. entitlements to assistance and citizenship rights more broadly), needs, views, and interests” (Hilhorst & van Wessel 2022, p. 112, see also Hilhorst et al 2021), is a key part of the humanitarian assemblage of activities in the 2020’s (Marcus & Saka 2006). Advocacy is a part of many organisations programming and output, and is a way of achieving (some) humanitarian goals, especially in the longer term. As Pupavac (2006, p. 256) noted, “Humanitarian advocacy was embraced as part of a fresh approach when the crises immediately following the collapse of the Berlin Wall cast doubt on traditional humanitarianism”, whilst Bridges (2010) sees that advocacy has been deployed both to try and mitigate or mollify the root causes of many crises, but also as a result of overall confusion about what ‘doing humanitarianism’ entails. From a more historical perspective, several scholars spotlight the relationship between humanitarian action and religious traditions of advocating on behalf of the poor or needy (Dromi 2020) and also the solidarity-based emotional link between people at large and the ‘distant suffering’ (Boltanski 2013).

### The state as a humanitarian actor

It has been widely recognized that humanitarian action had undergone a profound paradigm shift where the classical, Dunantist form of humanitarianism with emphasis on the humanitarian principles and centering the international aid system has not been replaced, but largely overtaken by a ‘resilience paradigm’ (Hilhorst 2018). Salient features of this paradigm are the recognition of crisis as interwoven with normality, a focus on the resilience of affected communities and downward accountability, and a more central role for local and national service providers. As part of the latter, there is now also more recognition of the role of states in humanitarian action.

As highlighted by Oscar Gomez (2025), for many decades the international humanitarian ‘system’ had scant respect for states, for a number of reasons. Firstly, (neo-)colonial assumptions about the capacity and indeed morality of a state in the ‘Third World’ reduced the state as a bystander, or gatekeeper, for the international humanitarians. Secondly, there is an assumption by aid providers that “embattled governments are unwilling to protect populations under threat” (2025, p. 49), perhaps due to the condescending assumptions noted before, but also perhaps due to the huge pressure a state faces as it undergoes a (humanitarian) crisis. Gomez then points out that these assumptions created a need for a “third party to play the humanitarian role” (*ibid*.), and so this space was filled by iNGOs, UN Agencies, and the Red Cross and Red Crescent movement. Per Paul Harvey and Jeremy Lind (2005), large aid bureaucracies are sometimes incentivized to highlight their key ongoing role in providing para-state services after a crisis and thus sideline the state, as a reasoning for their continued funding and existence. The role of the state remains central in contexts where recurring climatic-related hazard requires ongoing humanitarian response, such as recurring typhoons in the Philippines necessitating a well-developed and state-rooted humanitarian response mechanism (Baguio, 2025).

This is not to say that the state did not have a role in humanitarian responses of the last few decades, rather that this role was pushed out of the spotlight. In terms of the everyday machinery of a humanitarian response, state bodies and agencies are often active participants, from the creation and implementation of MOU’s that structure and facilitate the logistics or aid provision to granting permits that allow access or movement within a crisis context (Schattner-Ornan 2023). The institutional memory of a state-NGO relationship can help to smooth and ease coordination between military and humanitarian aid provider (Nahikian & Emmanuel, 2024). Meanwhile, state-associated national bodies including weather and climate institutes provide a backbone of data streams that can underpin Early Warning Early Action (EWEA) programming, pre-crisis interventions, or other risk mitigation actions carried out by humanitarian organisations (Jaime et al. 2022).

It is also important to note that states have a close relationship with how crises are brought about, especially as many humanitarian crises take place in contexts marked by conflict. In these situations, the warring parties are often treated with severe suspicion by humanitarian actors as interlocutors (see: Cunningham 2023), with humanitarian negotiations sometimes causing dilemmas around the need to speak and coordinate with the very state that is causing death and destruction (Healy and Cunningham 2023). Whilst it is necessary to continue to work with states in various ways during a conflict-related humanitarian crisis, humanitarian actors can even be drawn into suspicions of complicity in war crimes should they decide that continuing to work with a state is necessary for delivering aid (Ruamps 2023). Similarly, a state can become marginalized in humanitarian aid delivery and play only a token role, especially when a ‘weak’ state comes into contact with an international humanitarian machinery (ICVA 2021).

Within and during a disaster the social contract present in many states heaps expectation upon national or local governance structures to act to protect citizens (Ommer et al. 2024). Anticipatory action and risk-reduction in the face of climate change are understood as a state responsibility to protect citizens (O’Brien, Hayward & Berkes, 2009), and states join, ratify, and enshrine international Risk Reduction frameworks such as the Hyogo and Sendai Frameworks, as well as joining processes including the ‘Grand Bargain’ process prior to the 2016 World Humanitarian Summit. This was codified by the UN General Assembly in 1991 as being the “first and foremost” responsibility of the state itself (UNGA, 19 December, 1991, p50).

I see states as the linking pin between humanitarian work that happens across and throughout the disasters and crises, bridging the gaps between humanitarian response, rebuilding, and risk and vulnerability reduction.

#### Humanitarian diplomacy

Whilst this article focuses on humanitarian advocacy, it is important to consider that this happens in addition to more classical humanitarian diplomacy. Behind-closed-doors negotiations conducted in an official capacity and via established communications channels by professional diplomats, what is sometimes called ‘track-one diplomacy’, is accurately described as humanitarian diplomacy by various authors (Kirecci 2025), for example). Humanitarian diplomacy is formal: where a humanitarian, on behalf of an organization or several organizations, enters into political discussions and negotiations with a counterparty, in most cases a state (Egeland 2013; Rousseau & Pende 2020). The ICRC is a key Diplomatic Humanitarian actor (Rousseau & Pende 2020), enjoying significant diplomatic privileges, including observation status at the UN. Whilst Rousseau & Pende do highlight a difference between other international organisations and the ICRC (“it’s legal status is strictly functional”), they also point to Harrof-Tavel’s (2005, p.73) characterization of the ICRC as having a long tradition of confidentiality, and a “natural penchant for diplomacy and [being] behind the scenes”. To a certain extent, humanitarian diplomacy relies on the acceptance of the power and legitimacy of both the humanitarian imperative, and International Humanitarian Law (Regnier, 2011), mostly by states. Similarly, and as Turunen (2020, p. 467) points out, humanitarian diplomacy follows “idealistic logics”, which sets it apart from most other forms of ‘diplomacy’.

The study of humanitarian diplomacy often paints itself as a subsection or subfield of inter-state diplomacy (Bogatyreva, 2022, Minear and Smith 2007), and it relies on the claim of the humanitarian imperative, to allow humanitarian organisations *into the room* (Turunen 2020). As Bogatyreva (2022) highlights, the (once) widescale adoption of International Humanitarian Law can be seen as a victory of humanitarian diplomacy; seeking to crystalise the principles and outcomes of a successful diplomatic discussion, and anchored in state commitments. De Lauri (2018) defines humanitarian diplomacy as activities that seek to make “decision makers and leaders” act, which highlights that in many cases (as with most diplomacy), the discussions are high-level. Hilhorst & van Wessel (2022), meanwhile, present that humanitarian diplomacy is often focused on gaining access for humanitarian actors, as well as trying to persuade actors in high-intensity conflicts to respect International Humanitarian Law, especially at the senior level.

Humanitarian diplomacy traditionally leans on respect for International Humanitarian Law, implies a small group of senior interlocutors playing by established rules, and relies on the humanitarian imperative providing CSO’s and humanitarian organisations the ‘civic space’ to operate with relative freedom. However, these foundations are under severe strain, and in some cases have already crumbled.

### The rise of humanitarian advocacy

Humanitarian advocacy, in contrast, is what is often referred to as ‘track-two’ diplomacy, which is far more informal and unstructured interactions and discussions, especially involving non-state actors (Kirecci, ( 2025)). When these take place in public, or are addressed to a public audience, the interaction fully exits the diplomatic space. In simpler terms, humanitarian advocacy is what takes place outside of formal and often closed spaces of diplomacy, which is a growing amount of humanitarian interaction and negotiation. How has this shift come about? I argue that this is due to three interrelated factors: an erosion of respect for International Humanitarian Law, a shrinking civic space, and a widening in the number of stakeholders that are able to affect a humanitarian crisis context, for example Non-State Armed Groups (NSAG’s). Brought together, these three factors have helped to bring about a geopolitical environment where a large portion of interactions that intend to persuade or affect a humanitarian outcome come from different actors and take place in public, not according to established and formalized legal channels or frameworks, and that intend to shift public opinion rather than call on humanitarian stakeholders’ responsibilities as enshrined in IHL.

Looking at respect for International Humanitarian Law first, there is no doubt that armed conflicts in the 2010’s and 2020’s increasingly highlight the ease with which states can choose to ignore their obligations under International Humanitarian Law, for example with protecting civilians, proportional use of force, protection of vital infrastructure, or not targeting journalists. Egger (2023), for example, points to states simply waving away their obligations and/or crimes by claiming close compliance with International Humanitarian Law, and then even going so far as to call their armies “morally exemplary”. International Humanitarian Law has no single adjudicative body, which means that its enforcement is fragmented, and enforcement of International Humanitarian Law norms are incumbent upon states themselves (Tabak 2016, Tannenwald 2017). Some of these states see adhering to International Humanitarian Law as an infringement on their sovereignty (McLean & Hofman 2023), or simply prioritise other political or economic outcomes. In Israel’s multiple attacks on Gaza, for example, adherence to International Humanitarian Law has become secondary to political and security interests (Gunawan et al. 2025), and where bodies have stepped in to sanction government figures in relation to breaking International Humanitarian Law, countries have been reluctant to actually follow through and enforce arrest warrants on their territory. An example here is the reluctance of multiple countries to arrest Vladimir Putin for International Humanitarian Law violations in Ukraine (Ferdinandusse, 2025), or for even large countries like France and Germany declining to arrest the ICC-indicted Israeli Prime Minister Benjamin Netenyahu (Bussard 2024). This has left various humanitarian stakeholders in a state of powerlessness: unable to access the usual channels of humanitarian diplomacy, or trust that their privileges under IHL will be respected. Thus they turn to advocacy in public to try and affect humanitarian aid outcomes.

The past few decades have also seen a rise in the number of conflicts that include Non-State Armed Groups (NSAG’s), who are also often not included in formal humanitarian diplomacy talks, and must be persuaded in the advocacy space. For example, Munadi & Mena (2025) highlight Afghanistan as a humanitarian context where humanitarian negotiations take place on multiple levels at the same time, such is the fractured nature of territorial control in Afghanistan. In the same chapter, the authors highlight that what may have been approved at a ministerial level may still need to be discussed and persuaded at a local or regional level. Discussions and interactions in contexts such as Afghanistan cannot rely on IHL as a basis for diplomacy: it is unrealistic for a leader of an NSAG to comply with, for example, handing over subordinates that have broken International Humanitarian Law (Sassoli, 2010), or guaranteeing humanitarian access and protections and privileges for humanitarian and medical actors. As Ryngaert & Van de Meulenbroucke (2011) highlight, NSAG’s are subject to International Humanitarian Law, but have not often been involved in creating it, causing a gap between consent and adherence. NSAG’s often operate in a ‘grey zone’ with some backed by a state, as the Sudanese Rapid Support Force (RSF) is supported by the United Arab Emirates (Sayal 2025). The RSF has been accused of using water as a weapon of war (Geneva Academy of International Humanitarian Law Humanitarian Law & Human Rights, 2025), but as an NSAG its governance by International Humanitarian Law is questionable. Aside from discussions around the application of International Humanitarian Law to NSAG’s, the salient point is that humanitarian diplomacy, where it takes place between larger international humanitarian organisations and states directly, does not invite all of the stakeholders in a conflict to the negotiating table. This is particularly relevant with NSAG’s, as they can be militarily powerful parties to a conflict, but this highlights that a myriad of other groups, organisations, and polities with agency may be shut out of the direct discussions, whilst still be able to exercise their agency, via advocacy.

This collapse in respect for International Humanitarian Law combines with other pressures to create a ‘Shrinking Space’ for humanitarian and civil society operations (Collinson & Elhawary, 2012; Roepstorff, Faltas & Hövelmann, 2020). Whilst the collapse in International Humanitarian Law prevents operations and responses of humanitarian CSO’s by denying a semblance of protection in conflict, its domestic counterpart is the suspicion or outright hostility of governments towards (humanitarian) CSO’s. This could be through requiring punitive registration practices and a requirement to refuse any international funding, as in the case of Hungary (Buzogany et al., 2022), a requirement to publicly refer to themselves as a ‘Foreign Agent’ thus eroding public trust in the organization, as is the case in Hungary, Poland, and Russia (Ploszka, 2020), or even arbitrary listing of CSO’s, including humanitarian and relief organisations, as ‘terrorist organisations’, as has happened in Israel for Palestinian organisations post-2024 ceasefire (Zorob & Paluszek 2025). All in all, and as Ploszka, 2022) highlights, in 2018 citizens in more than 111 countries around the world faced onerous barriers to setting up, registering, or operating CSOs.

These currents have come together to create an environment where a large number of humanitarian interactions, whether between states, or between states and NGO’s, or between NGO’s and non-state parties such as NSAG’s now take place outside of the rules-based and formal humanitarian diplomacy space. Thus the relative importance of humanitarian advocacy has grown.

### Humanitarian advocacy and resilience humanitarianism

There are several parallels between the rise of a ‘resilience humanitarianism’ paradigm (Hilhorst 2018) and the relative rise of humanitarian advocacy. ‘Resilience humanitarianism’ has arisen during a wave of more rights-based approaches to humanitarian action and development at large (See: Gauri & Gloppen 2012, Ouyang et al., 2012, Pietropaolo 2016, Borgrevink & Sandvik, 2022) which requires more emphasis on advocacy as a way to try and transform social conditions that have led to a crisis occurring. This is referred to again by Bradley (2022) as a brand of ‘political’ humanitarianism, which eschews the strict neutrality in the ICRC style where humanitarian organisations do their work ‘behind the scenes’ or indeed stay ‘above’ politics, and instead embrace protecting and trying to advocate for people’s rights, both political and socio-economic. Similarly, humanitarian advocacy fits into an understanding of crisis as being part of a longer term process of vulnerabilities, exposure, and hazards (Blaikie et al. 2014), as well as the integration of peacebuilding and development work, which Mena calls “Nexus Thinking” (2025).

Related to this is the growing relevance and appreciation of the Humanitarian, Development, and Peacebuilding ‘Nexus’ (See Weishaupt 2020; Mena, Brown & Ji, 2022; Joireman & Haddad 2023; Hegertun et al. 2023; Mena 2025). The HDP Nexus, which has a somewhat disputed definition, nonetheless refers to a number of initiatives post the 2016 World Humanitarian Summit that seek to link and build coordination and mutual support between Humanitarian, Development, and Peacebuilding activities – recognizing that they are often interwoven. Undoubtedly, ‘resilience humanitarianism’ as a style of doing humanitarian work is part of this mosaic, as are rights-based approaches to humanitarianism, altogether (and in some cases overlapping) forming a very wide range of groupings and organisations that contribute to humanitarian crisis response, preparedness, or mitigation, depending on the context or nature of the crisis.

These various currents have come together to build a form or style of humanitarianism that is politically aware (if not always engaged), sees disaster as a result of the interaction of hazards with social and political vulnerabilities and (lack of) capacities (Hilhorst & Bankoff 2022), and that is aware of the need to engage with a wide range of people, groupings, communities, organisations, polities, and so on (see also: Richey 2018). Thus achieving humanitarian goals becomes more of a persuasive task: especially as humanitarian needs constantly outstrip humanitarian capacities. At the same time as these currents have reformed how humanitarianism is done from within and below, forces outside of and above the world of humanitarian action have eroded some of the pillars that humanitarian diplomacy rests upon.

In any case, the growth of the ‘resilience humanitarianism’ (Hilhorst 2018) brand of humanitarian action also highlights that a much wider range of stakeholders need to take part in aid negotiations, with Hilhorst referring to the rise of the “humanitarian ecosystem”, rather than simply ‘system’. With such a wide range of stakeholders taking part in negotiations, and with the widely varying relative power of these organisations, groupings, and polities, humanitarian diplomacy in the traditional sense now takes place as part of a broader negotiation process across broader levels. Similarly, humanitarian advocacy can be performed more or less by all actors associated with a humanitarian context, including affected communities, groupings, and local/national organisations. Thus the relevance of humanitarian advocacy in helping to shape overall negotiations grows.

### States as advocates instead of diplomats

With fewer state parties to conflicts (in particular) still ‘playing by the rules’ and keeping to legal agreements, far more humanitarian negotiations are conducted based on softer premises. Indeed, Donald Trump recently highlighted that his own personal morality is more of a guide to his actions than international law: ‘“It’s the only thing that can stop me” (Yang, 8 January 2026). Less powerful states, through relative international “smallness” must undertake advocacy and coalition building through advocacy to achieve their goals in the international arena (Cunha & Thorhallson, 2023), including advocating for humanitarian outcomes and even adherance to International Humanitarian Law.

Per Kaiser & Rochefort (2007), in the second half of the twentieth century there has been the rise of “big powers”, especially the United States under the ‘pax Americana’—in some cases a fig leaf for neo-imperial aspirations (Cohrs 2018). Nevertheless, the US has often been the guarantor of ‘the rules-based international order’ (Trachtenberg 2025) in the 21 st Century. Under the second Trump administration this role is being relinquished and exchanged for ‘America First’ policy directives that prioritise bilateral dealmaking, a distrust for multilateralism, and logics of hard economic and military power (Cooley & Nexon 2025). Few other large powers are rushing to fill this role.

Pressure on the UN, for example, has seen the launch of the ‘UN80’ reform agenda, which seeks to be “a system-wide push to streamline operations, sharpen impact, and reaffirm the UN’s relevance for a rapidly changing world” (UN News, 23 June 2025). The rise of powerful states acting independently has created cracks in the effectiveness of the Security Council as a body, too. After a short-lived period following the fall of the Berlin Wall, when the Security Council became more effective, renewed cracks grew deeper during the Arab Spring; especially NATO’s 2011 Libya bombing campaign, the Syrian Civil War from 2014, and the various Russian incursions into Ukraine throughout the 2010’s (von Einsiedel, Malone & Ugarte, 2015). Paralysis at the Security Council has only grown, with countries very willing to use their veto power to stop even condemnation of what they or their allies are doing. Examples include Russia’s veto-ing of action in Syria, or the USA veto-ing action or condemnation of Israel. The Security Council has also increasingly blocked initiatives backed by smaller countries (i.e., countries not on the Security Council). Referring to the invasion of Ukraine by Russia in 2022, Brunk & Hakimi (2022) note that “the Security Council also considered a draft resolution, submitted by eighty-two countries, that would have condemned Russia’s aggression and called for an immediate ceasefire in Ukraine. That resolution failed on account of Russia’s veto”. Similarly, in 2025, 149 countries backed a General Assembly resolution demanding that Israel lift its humanitarian blockade on Gaza: “as a result of the Security Council’s failure on 4 June to adopt a resolution on the matter, due to a veto by the United States” (UNGA, 12 June 2025). At the same time, the relationship between China and the United States has been deteriorating, further weakening the role of the UN Security Council.

Other multilateral institutions have also become less relevant compared to bilateral relations; including the World Trade Organisation, the G20 (Nageswaran et al. 2025), and the organisations created around the Paris Climate Agreement (Bello 2021). Whilst it may be unrealistic to portray an ever-declining trend for multilateralism in the current world, as put by Hanhimaki (January 20, 2025): “the idea of an ever-expanding liberal international system built upon the benevolent backing of the United States is far from being realised… a credible and coherent alternative fails to emerge”.

In this geopolitical milieu, smaller states interventions in humanitarian crises, especially calls for adherence to International Humanitarian Law, no longer look like humanitarian diplomacy. Decisions are taken on an increasingly small scale, often unilaterally or bilaterally, and institutions such as the UN General Assembly are unable to pass independent proposals without Security Council approval. However, as will be approached in the next section, multilateral bodies such as the UN General Assembly do provide stages upon which states and their leaders can try to carry out humanitarian advocacy through persuasion, mobilization, and denunciation.

## Humanitarian advocacy as a state: analysing the advocacy actions of Slovenia and Colombia

The case studies chosen for this analysis are the Republic of Colombia, and the Republic of Slovenia, in the context of the Israeli War on Gaza from October 7, 2023. At first glance, the two may not have much in common: one being located in Latin America, with a population of over 50 million people, and other being located in Central Europe, with a population of around 2 million people. Both countries have been extremely vocal during the course of the developing Israeli genocide in Gaza, and have taken their calls for humanitarian outcomes into the public space. Various other countries have also carried out a range of humanitarian advocacy activities, both related to Palestine, and related to other humanitarian crises in the 2020’s, including Spain and South Africa in the case of Gaza, but also Kenya and Zimbabwe’s leaders in the case of the DRC/M23 conflict and humanitarian crisis in Eastern DRC (Office of the President of the Republic of Kenya February 8, 2025). These various other countries’ humanitarian advocacy efforts would be an interesting path for further research, as would an analysis of humanitarian advocacy by the United States in a historical sense, or by leaders of states with a markedly religious character, for example the Vatican City.

### Methodology

To choose the sources for analysis, I chose to focus on official state communications in the form of speeches given by President Gustavo Petro (Colombia, elected 2022) and President Natasa Pric Musar (Slovenia, elected 2022), including where other government figures have ‘stood in’ for the Presidents – for example Slovenian Prime Minister Robert Golob (elected 2022), as well as official state press releases. Sources were included only if they referred to actions, speeches, or policies that had taken place after the beginning of Israel’s retaliatory action after October 7, 2023, and had been made by politicians speaking in an official capacity, until the closing date of data gathering in January–February 2026. The initial dataset was gathered via humanitarian and Gaza-related keyword searches on the web portals of the office of the President of Slovenia and the office of the President of Columbia. To fill any gaps, I then searched through the speeches of both Presidents at multiple UN General Assembly sessions since the beginning of 2023 as part of the “General Debate”. Finally, the same process was repeated via the press office web archives of the European Union (including the European Parliament and European Commission), for Slovenia, and the Organisation of American States for Columbia. Finally, to further substantiate the source list, keyword searches were carried out via the portal of the Associated Press and Reuters, and via the official press offices of the Republic of Slovenia and the Republic of Columbia in order to also highlight policy and press releases from the countries that are examples of humanitarian advocacy, but were not announced by President’s speeches. Where necessary, statements were translated into English using machine translation software. This resulted in an initial source list of 42 speeches, press releases, and one op-ed by President Petro in the Guardian newspaper, 23 documents were from representatives of Slovenia, and 19 from Colombian ones. Deeper reading reduced this to 36 sources in total, with the extra 6 excluded for being less relevant to the question, for example only making passing mention of International Humanitarian Law in the title, or for when speeches were duplicated in press office listings.

To create a code for further analysis, I utilized a modified typology of advocacy in public health, devised by Earnest et al. (2023), which focuses on the “domain of influence”, or audience, the type of advocacy skill on show (the form or mode), and the intended societal level of change. This approach is influenced by a wish to analyse the relationships between the different moving parts of each humanitarian advocacy context, and so, after reading the sources with this initial code in mind, I integrated Slim & Bornwick’s pithy functions of advocacy as being intended to inspire “persuasion, mobilisation, denunciation” (2005, p84). There are a wide range of theoretical explanations for how and why advocacy efforts are able to affect policy (Burstein 2021), with a majority of them agreeing that advocacy efforts in the main are able to influence policy choices in a range of governance systems. With the three groupings now tentatively noted as ‘Public Advocacy’, ‘Legal Advocacy’, and ‘Policy Advocacy’, I again re-read and highlighted the sources with these categories as the ‘code’.

‘Public Advocacy’ is aimed at a public audience, or society at large, and intended to persuade or draw people to a narrative. ‘Legal Advocacy’ is strongly related to International Humanitarian Law and traditions of humanitarian diplomacy, and often has a strong denunciation element, and ‘Policy Advocacy’ is intended to mobilise empowered institutions or groupings by setting an example by carrying out some form of action. These actions, as will be approached, often involve economic or socio-political sanctions, which may not have a particularly forceful effect in and of themselves, but are powerfully symbolic.

### Public advocacy

‘Public Advocacy’ derives, in my view, from the human rights and campaigning background of several expressions of humanitarianism (Bridges 2010, Metcalfe-Hough 2021; Hilhorst & van Wessel 2022), and includes a strong element of ‘speaking truth to power’, which may be interrelated to its historical basis in rights-based critiques of development in the mid-20th Century, and the implied economic and social rights of various international human rights legislation (Nelson & Dorsey 2008a). It often intends to use the power of shame (Bradley 2022). However, it often stems from a form of moral outrage, and a compulsion so ‘speak out’ (ibid). In trying to bring public sentiment towards a particular focus, ‘Public Advocacy’ might use Manichean overtones, stylized or emotionally-driven portrayals, and calls to action that present a potential or simplified ‘solution’ to the subject of focus (Saez & Bryant, 2023). It might also include elements of temoignage, or witnessing and wishing to transfer the voices of the suffering (Patel, et al. 2025), but does not necessarily imply acting upon what has been witnessed (Gorin 2021). It can be considered divisive (Slim & Bornwick, 2005). From the sources utilized for this analysis, 33 of the 36 sources exhibited some form of ‘Public Advocacy’, whether due to the place they were delivered, the emotive and moralistic language used, or the stylized portrayals utilized.

Public pronouncements by Presidents/Prime Ministers on behalf of their nation form an important component of Public Advocacy, for example President Petro of Columbia’s speeches as the UN General Assembly General Debates: “The control of humanity based on barbarity is under construction, and Gaza and Lebanon are its prime examples. When Gaza dies, all of humanity will die” (Petro, 24 September, 2024). Throughout this speech, President Petro quite clearly utilizes a strongly moralistic tone, which also invokes strongly religious imagery: “It turns out that God's people were not the people of Israel, nor the people of the United States of America, but rather all of humanity, and the children of Gaza, that was humanity, God's chosen people.” (Ibid.). In his UN General Assembly address the following year, President Petro also shows how Public Advocacy can be confronting and make for uncomfortable listening, stating that “This place [the General Assembly] is a silent witness, and accomplice to genocide in today's world” (Petro, 23 September, 2025).

In her speech on the same day, President Musar gave explanations for why the post-Cold War period had not resulted in a “vision of lasting peace, security and co-operation as enshrined in the UN Charter” (Musar, 23 September, 2025), highlighting that “international law appears to stand at the precipice of irrelevance” and that “the Genocide Convention risks becoming a relic of the past” (Ibid.). Though using less emotive language than President Petro, President Musar does strike a strong tone of moral outrage, saying that “Worse still, hunger is now being weaponised – turned into a tool of war! We receive reports on a daily basis of how women and children are starved because they are marked as legitimate targets, as enemies. How cruel, how inhumane, is that, ladies and gentlemen?” (Ibid.). Similarly, in 2024, Slovenian Prime Minister Golob ended his address with an emotive call directly addressed to the Israeli Prime Minister of “stop the bloodshed, stop the war, stop the occupation of Palestine!” (Golob, 27 September 2024).

Public advocacy may be intended to draw the attention of powerful stakeholders, or inspire a critical mass of public opinion as a way of changing geopolitical reactions to humanitarian crisis.

### Legal advocacy

‘Legal Advocacy’, as mentioned, utilizes the structures and weight of International Humanitarian Law. Examples of this type of advocacy are often laden with notions of formal justice, appealing to International Humanitarian Law as the embodiment of natural justice, and due to its use of international law apparatus, is also quite legalistic in its stylistic expression – in many cases being devoid of emotional language, matter-of-fact, and couched in formal terms. As discussed, the lack of an overarching single organization for the enforcement and maintenance of International Humanitarian Law can make flouting International Humanitarian Law easier, however ‘Legal Advocacy’ can aim to redress this by providing a grouping that promotes norms (Keck & Sikkink 1998), whether they are formally codified, or not. I use ‘Legal’ in the widest sense here, so as to include formal legal action in the manner of ‘strategic litigation’ or where a rights or legal violation is brought before a court as part of a wider initiative to achieve a goal, in many cases bringing the issue to the public’s attention, or increasing its salience in political discourse (Van Der Pas 2021), but also in terms of ‘legal mobilisation’, where legal structures are used as part of a wider law-based civic advocacy campaign for social justice ends (Handmaker 2019; Buckel, et al. 2024).

Within the documentation and actions analysed for this article, the creation of ‘The Hague Group’ is particularly important: as a grouping of countries coalescing around protecting international law in the case of the Israeli military campaign in Gaza, but with a public advocacy goal aside from the strategic legal actions taken at the International Court of Justice. At the emergency meeting of The Hague Group’s members in Bogota, July 2025, its official statement highlights the group’s goals in legal terms: “In the deliberations at the Bogotá conference, all 30 participating states unanimously agreed that the era of impunity must end— and that international law must be enforced without fear or favour through immediate domestic policies and legislation” (The Hague Group, 16 July 2025). Slovenia’s signing of a Memorandum of Understanding with the ICRC at the Bled Strategic Forum in September 2025 is also symbolic advocacy act rooted in the traditions of formal justice and International Humanitarian Law, with the Republic of Slovenia also leveraging its temporary position on the UN Security Council to promote International Humanitarian Law adherence (ICRC, 2 September 2025). Similarly, Slovenia moved to ban several Israeli government officials, including the Prime Minister, from entering Slovenia due to the indictment of those officials at the International Criminal Court (AP, September 26, 2025). Other actions through international justice mechanisms are notable here, for example Columbia’s depositions during the initial advisory hearings around Israel’s conduct in Gaza that took place in late 2024 (Presidencia.gov.co, February 21, 2024). There is often an overlap between actions that typify Legal Advocacy and Public Advocacy. In some cases it may be that legal routes are employed to build a bigger stage for Public Advocacy efforts, for example though strategic litigation practices, that may also be useful in upholding International Humanitarian Law in general (Strobel 2025).

Similarly, the (legal) recognition of the State of Palestine “as a precursor to a stable two state solution” (Pirc Musar, 21 May 2025) is a legal step that has both a ‘Policy Advocacy’ and ‘Public Advocacy’ dimension, with the Slovenian government’s press office highlighting that by recognising a Palestinian state it hopes to inspire other states to take the same step (Government of the Republic of Slovenia, 5 June 2025). Smaller countries utilizing their convening power within multilateral institutions has elements of all three categorisations mentioned. For example, during the course of data collection for this article, Slovenia took up its seat as the Chair of the UN Security Council, for two month-long periods in September 2024 and then again in December 2025. The Slovenian government highlighted its wish to keep the unfolding genocide in Gaza, as well as illegal settlement building in the Occupied Palestinian Territories “on the Council’s agenda” (Government of the Republic of Slovenia, 1 December 2025). It also highlighted its role in bringing about a Security Council Resolution on calling for a full ceasefire in Gaza, and a release of Israeli hostages (Government of the Republic of Slovenia, 11 June 2024). This blurs the lines between public and legal advocacy techniques – as does President Petro’s use of his public platform to renew his call for parallel peace processes in Ukraine and Palestine at the UN General Assembly in September 2025 (UN News, 23 September 2025).

### Policy advocacy

Meanwhile, ‘Policy Advocacy’ can be seen as unilateral policy actions carried out by states that have less economic, political, or military power as a way of establishing their own character (i.e. by acknowledging the post-colonial character of a state by standing up for the rights of states being affected by coloniality (Ottaway, 2002), or as a way of building alliances that can make up for “smallness” (Cunha & Thorhallson, 2023), or as a way of building networks with other like-minded states that might be small or middle powers (Cooper & Flemes 2013). The key element within ‘Policy Advocacy’ is that a state’s actions (for example suspending a trade agreement) are unlikely to be drastically effective in economic or military terms, rather it is the symbolism of the policy that gives it pertinence. The US, for example, would not typically carry out ‘Policy Advocacy’, as its policy choices have a direct impact thanks to the US’ relative economic, political, and military power: there is less of a need to persuade! Policy Advocacy can be seen as the implementation of ‘Public Advocacy’, in that it entails states deploying their policymaking capacity for the purpose of making a conceptual or moralistic position tangible. Nelson & Dorsey (2008a, p. 104) note three characteristics for assessing how development NGO’s deploy rights-based approaches, which are useful parallels for considering when a state’s humanitarian advocacy approach fits into this category: “How public and visible, how system-wide, and how clearly and specifically rooted in internationally recognized human rights standards and principles”.

‘Policy Advocacy’ actions are often unilateral but can then develop into multilateral initiatives – for example in the case of Colombia suspending coal exports and new military equipment imports with Israel, as well as suspending the free trade agreement between the countries, and finally severing diplomatic ties (Suarez, May 3 2024; Rueda, June 9 2024). It should be noted here that the states of Bolivia and Belize also severed their diplomatic ties with the state of Israel at a similar time, which may be an example of multilateral or at least parallel action being taken by smaller countries as a way to broaden the impact of each country’s individual policy choice.

Similarly, Slovenia banned the import, export, or transit of weapons to or from Israel in July 2025; it was the first European Union member state to do so (Zerdin, August 1, 2025). An arms transfer researcher from the Stockholm International Peace Institute refers to the policy change as symbolically important, rather than materially important (Ibid.). Other examples of Policy Advocacy in this example include the recognition of the State of Palestine, or banning imports of products made on occupied Palestinian land in the West Bank (Reuters, August 6, 2025).

Considering the three characteristics mentioned earlier (Nelson & Dorsey 2008a), Slovenia’s various policy positions around banning weapons shipments, transfers, and export, combined with its ban on goods made in the occupied West Bank, and its declaration that two far-right, settler Israeli politicians, Itmar Ben Gvir and Bezalel Smotrich, are personae non grata in Slovenia’s territory (de la Baume, 1 August 2025) are indeed systematic, public, and couched in the language and principles of international human rights: when declaring these politicians as persona non grata, the Slovenian government’s spokesperson specifically drew attention to the two Israeli minister’s “genocidal statements”, calls for violence against Palestinians, and calls to expand Israeli settlements that are illegal under international law (Government of the Republic of Slovenia, 17 July 2025). In some cases, ‘Policy Advocacy’ initiatives can overlap with ‘Public Advocacy’ initiatives, for example in the Guardian article by President Petro “Governments like mine have a duty to stand up to Israel. Far too many have failed” (8 July 2025). In this article, President Petro utilizes the emotive language of ‘Public Advocacy’: “Over the past 600 days, the world has watched Benjamin Netanyahu lead a campaign of devastation in Gaza…”, whilst also highlighting various policy initiatives as examples for potential emulation, highlighting both Colombia’s suspension of coal exports with Malaysia’s banning of Israeli-flagged cargo ships from docking in its ports.

It is important to also note some of the limitations of these categories of advocacy practices: many advocacy actions by states cross these category boundaries, or indeed change their categorization within these boundaries. An example here is the creation of the afore-mentioned ‘The Hague Group, which specifically notes legal action being undertaken by the state of South Africa at the ICJ around seeking a ruling on Israel’s actions in Gaza (The Hague Group, 31 January 2025), but is also a way for several countries with lesser relative power to join together as an alliance that can indeed make up for their relative “smallness”. Whilst these categorisations have been conceived as useful ways to look at and analyse how countries do advocacy, I would caution against seeing them as closed or discreet packages.

## Conclusion, limitations and implications

This article has sought to identify how states carry out humanitarian advocacy, itself a trend that has arisen from studies of humanitarian diplomacy. This article helps set a basis for further research in these areas: the first on the methods and roles of multiple actors within wider humanitarian advocacy dialogues; the second on the nature of who makes decisions within humanitarian contexts, and how; and the third on studying the actual impact of the three forms of advocacy.

This study does of course have its limitations. Research into humanitarian advocacy itself is developing, and not yet hugely widespread, meaning that there are significant gaps within our knowledge about specifics of how humanitarian advocacy is done by various actors, and how effective it is. Similarly, the role of the state as a humanitarian advocate is not fully understood in a wide range of contexts, though there are various initiatives in this area (e.g., Baguoi 2025). In methodological terms, analysing the selected documents may be limited by a reliance on the governments of Colombia and Slovenia’s own communications offices, and what they choose to make publicly available. It is also good to highlight here that whilst Colombia and Slovenia have been vocal in their humanitarian advocacy around the ongoing genocide in Palestine, other states may be more informative for researchers when looking at other humanitarian crises. It is my hope that this article can add a voice to discussions on the role of states as humanitarian advocates, and provide a basis for further work on the subject.

The implications of studying states as humanitarian advocates, and the categories proposed is to spark further discussions around why a state might choose to (for example) engage in “Policy Advocacy” as opposed to “Legal Advocacy”, or “Public Advocacy”, or to lay the groundwork for further analysis of the different modes of humanitarian advocacy activities carried out by states. As Humanitarian Studies continues to move its focus onto affected populations, and national groupings, organisations, and government institutions, it is important to highlight the broadening of who is involved within humanitarian negotiations, dialogues, communication, and advocacy – and to what ends.

## Data Availability

No datasets were generated or analysed during the current study.
